# A study of diabetic ketoacidosis in the pregnant population in the United Kingdom: Investigating the incidence, aetiology, management and outcomes

**DOI:** 10.1111/dme.14743

**Published:** 2021-11-28

**Authors:** Caroline Diguisto, Mark W. J. Strachan, David Churchill, Goher Ayman, Marian Knight

**Affiliations:** ^1^ National Perinatal Epidemiology Unit Nuffield Department of Population Health University of Oxford Oxford UK; ^2^ Pôle de gynécologie obstétrique, médecine fœtale, médecine et biologie de la reproduction Centre Olympe de Gouges CHRU de Tours Université de Tours Tours France; ^3^ Université de Paris CRESS INSERM INRA Paris France; ^4^ Metabolic Unit Western General Hospital Edinburgh UK; ^5^ Research Institute in Healthcare Science University of Wolverhampton The Royal Wolverhampton Hospital NHS Trust Wolverhampton UK

**Keywords:** diabetes, incidence, ketoacidosis, population‐based study, pregnancy, stillbirth

## Abstract

**Aim:**

To estimate the incidence of diabetic ketoacidosis (DKA) among pregnant women, describe its clinical features, management and outcomes and identify the risk factors for the condition.

**Methods:**

A national population‐based case–control study was conducted in the UK using the UK Obstetric Surveillance System between April 2019 and September 2020 including all pregnant women with DKA irrespective of the level of blood glucose. The incidence rate of DKA in pregnancy was estimated. A case–control analysis limited to women with type 1 diabetes was performed comparing characteristics of women with DKA (cases) to those of women whose pregnancies were not complicated by DKA (controls).

**Results:**

In all, 82 women were identified with DKA in pregnancy; 6.3 per 100,000 maternities (95% CI: 5.0–7.9). No maternal deaths occurred, but perinatal mortality was 12/73 (16%) with 11 stillbirths and one neonatal death. DKA episodes mostly occurred in women with type 1 diabetes (85%) and in the 3^rd^ trimester of pregnancy (71%). Episodes were mainly precipitated by infection (21%), vomiting (21%), steroid therapy (13%) and medication errors (10%). Fifteen percent of women had more than one episode of DKA during their pregnancy. Risk factors associated with DKA among women with type 1 diabetes identified through the case–control analysis were the woman and/or partner not being in a paid employment and having at least one microvascular complication of diabetes before pregnancy.

**Conclusion:**

DKA in pregnancy was associated with high perinatal mortality and was linked with factors related to socio‐economic deprivation, mental health problems and long‐term difficulties with glycaemic control.

**What is already known?**

- DKA in pregnancy is rare and carries a risk of morbidity and mortality for the pregnant woman and the fetus

**What this study has found?**

- The incidence of DKA in pregnancy was 6.3 per 100,000 maternities
- Half of the DKA episodes were euglycaemic
- No maternal deaths occurred, but perinatal mortality and rates of preterm births were high
- 15% of women with DKA episodes had more than one episode in their pregnancy

**What are the implications of the study?**

- Particular attention should be paid to women at high risk of DKA; guidelines are needed on appropriate management following episodes of DKA, given the high risk of recurrent DKA, preterm birth and later stillbirth.

## 1 INTRODUCTION

Diabetic ketoacidosis (DKA) is characterised by hyperglycaemia, hyperketonaemia and metabolic acidosis. It is the result of insulin deficiency and is a medical emergency that carries risks of morbidity and mortality in the pregnant and non‐pregnant population. Single‐centre studies in the 1990s reported the incidence of DKA in pregnancy to be between 0.2 and 3%.^1^, ^2^, ^3^ More recent data from the National Pregnancy in Diabetes Audit (NPID) in 2016 suggested that 2.7% of pregnancies among women with type 1 diabetes were complicated by DKA in the UK, but it is increasingly recognised that DKA may occur in other types of diabetes. The true incidence of DKA in pregnancy is unknown and particularly needs to be investigated since the prevalence of diabetes (types 1 and 2) in the reproductive population is increasing.^4^, ^5^, ^6^, ^7^

DKA in pregnancy remains a poorly understood condition as data regarding risk factors, clinical features and outcomes of DKA in pregnancy are scarce and are limited to case series,^8^, ^9^ retrospective studies^3^ and review articles.^10^, ^11^, ^12^, ^13^ One of the peculiarities of DKA in pregnancy is that it can occur at near normal levels of blood glucose (euglycaemic DKA).^14^, ^15^, ^16^, ^17^ The management of DKA in pregnancy is not specifically addressed in national guidelines and it is unclear how guidance on the management of DKA in the non‐pregnant population should apply to pregnant women.^18^ Regarding outcomes, DKA carries risks for both the pregnant woman and the fetus.^10^, ^11^ Fetal and neonatal morbidity can be the direct consequence of the extremely poor tolerance of the fetus to acidosis, which can lead in the most severe cases to intrauterine death or preterm delivery for maternal compromise. DKA may be a rare complication, but because of its serious consequences for pregnant women and their babies, accurate information about DKA in pregnancy is needed from adequately powered studies to inform women and healthcare professionals.

The aims of this study were to estimate the incidence of DKA in pregnancy in the UK, describe its clinical features, management and outcomes and to identify the risk factors for this condition.

## 2 METHODS

### 2.1 Design

A population‐based matched case–control study was designed using the UK Obstetric Surveillance System (UKOSS), a research platform that collects national population‐based information on specific severe pregnancy complications. All 194 maternity units in the UK participate in UKOSS and each has designated UKOSS clinician reporters who are sent monthly notification emails containing a list of conditions under surveillance and are asked to report the number of women with these conditions or to confirm zero cases; the UKOSS administration is set within the National Perinatal Epidemiology Unit at the University of Oxford.

For the purposes of this study, data on women affected with DKA while pregnant (cases) and on pregnant women with diabetes who did not have DKA (controls) were collected between April 2019 and September 2020. The cases and controls were defined as follows:

### 2.2 Cases

Any pregnant woman, with any type of diabetes, who was managed for DKA, irrespective of the level of blood glucose.

### 2.3 Controls

Controls were identified from the pregnant population with diabetes giving birth in all hospitals in the UK. Maternity units were requested to send information on the next woman with diabetes giving birth in their unit at two time points during the study. At the first time point (November 2019), units were requested to provide details of the next woman giving birth with any type of diabetes. Because the number of cases of DKA in women with type 2 and gestational diabetes was very small, the case–control analysis was focused on women with type 1 diabetes. At a second time point (November 2020), units were requested to provide details of the next woman giving birth with type 1 diabetes in order that sufficient controls were identified to ensure at least one control per case, matched for type 1 diabetes.

### 2.4 Data collection and consent

Information about each patient was collected by the local UKOSS reporting team after the episode of DKA meaning that the clinical management of the woman was not influenced by the data collection in any way. Women's demographic details, previous obstetric and medical history, information on the ongoing pregnancy, birth and infant outcomes were collected. Birth outcomes included the mode of birth and the indication for a caesarean section, if applicable. Indications for caesarean sections were classified as (a) ‘maternal compromise’ when the caesarean was performed for a severe maternal condition that could be related to DKA or another complication in pregnancy; (b) ‘fetal compromise’ where caesareans were performed for a non‐reassuring fetal status necessitating immediate delivery and (c) ‘other’ when the caesarean had been performed for any other indication. Neonates were defined as Small or Large for Gestational Age when their birthweight was below the 10^th^ or above the 90^th^ centile according to the Intergrowth birthweight references which are adjusted for gestational age of birth and sex.^19^ For cases of DKA, the causes or precipitating factor, clinical features, management and outcomes for the mother and baby were collected. Anonymised information was entered on data collection forms and securely transferred from hospitals to the central UKOSS database hosted on secure servers at the University of Oxford. Individual consent was not required for collection of anonymous data.

### 2.5 Sample size

One of the aims of the study was to estimate the incidence of DKA during pregnancy. Assuming 700,000 women give birth annually in the UK and that approximately 2.5% of women have pre‐pregnancy diabetes, with an incidence of DKA of between 0.5% and 3%, the estimated range of the number of cases of DKA in pregnancy was 87–525 per annum. The National Diabetes in Pregnancy audit, which collects data on pregnant women, set the incidence much lower at 0.41%.^20^ Using this figure, the number of cases of DKA during pregnancy was estimated to be between 28 and 87 cases per annum. It was felt, based upon clinical experience, that this lower range was a more realistic estimate and that a period of data collection of 18 months would yield a sufficient number of cases of DKA for meaningful analysis.

### 2.6 Statistical analysis

The incidence rate of DKA in pregnancy (number of DKA cases in pregnant women/number of women giving birth over the same time period across the UK) and its 95% confidence interval (CI) were estimated. Denominator data were obtained from routine statistics published by the Office for National Statistics (For England and Wales), National Records Scotland and the Northern Ireland Statistics and Research Agency and the national Diabetes in Pregnancy Audit. A sensitivity analysis was carried out estimating the incidence using the definition of DKA applied to the non‐pregnant population: women with an arterial pH <7.30 and/or a bicarbonate level <15 mmol/l and capillary ketonaemia of >3 mmol/l or ketonuria of >2 pluses on dipstick testing. The incidence of ‘euglycaemic DKA’, defined as a venous blood glucose level <14mmol/L, was also estimated.

Clinical features of the women with DKA and the individual DKA episodes (timing, causes, clinical features, management and outcomes) were documented.

A matched case–control analysis was performed comparing demographic characteristics, delivery, maternal and neonatal outcomes of women with type 1 diabetes who had DKA (cases) to those of women whose pregnancies were not complicated by DKA (controls).

We also conducted an exploratory multivariable analysis using logistic regression to identify factors associated with DKA in women with type 1 diabetes. We adopted the perspective of a physician aiming to identify pregnant women at risk of DKA and therefore limited this analysis to pre‐pregnancy and pregnancy variables selected by Directed Acyclic Graphs. Analyses were performed using STATAv13 (StataCorp).

## 3 RESULTS

Between February 2019 and November 2020 (22 months recruiting period), 83 women were notified with DKA in pregnancy in the UK, one of which was a duplicate report. Cases were reported from 65 units (49 units reporting one case, 15 units reporting two cases, one unit reporting 3 cases and 129 units confirming zero cases). Cases were predominantly in women with type 1 diabetes with much smaller numbers in women with type 2 and gestational diabetes. The estimated incidence of DKA in pregnancy was therefore 6.3 per 100,000 maternities (95% CI: 5.0–7.9). Forty‐seven of these women (57%) met the definition of DKA that applies to the non‐pregnant population and the incidence of DKA in pregnancy using this definition was 3.6 per 100,000 maternities (95% CI: 2.7–4.8). The incidence of euglycaemic DKA was 3.0 per 100,000 maternities (95% CI: 2.1–4.1). When considering only women with type 1 and type 2 diabetes, the incidences of DKA are estimated to be 16.6 (95% CI: 13.0–20.9) per 1000 women with type 1 diabetes giving birth and 1.1 (95% CI: 0.4–2.5) per 1000 women with type 2 diabetes giving birth.

No maternal deaths were reported and 11 stillbirths occurred in the cohort of women with DKA; seven occurred at the time of DKA and four occurred later in pregnancy (in women who did not have further episodes of DKA). Three of the later stillbirths occurred at 35‐ to 37‐weeks gestation in women who had their DKA in their 2^nd^ trimester. The fourth stillbirth occurred at 25 weeks in a woman whose DKA episode was in the first trimester. One preterm neonate died due to complications of prematurity after an emergency delivery in the early third trimester for fetal compromise during the DKA episode. Overall, perinatal mortality was 12/73 (16%).

### 3.1 Maternal features

Most DKA episodes occurred in women with type 1 diabetes (70/82; 85%), but episodes in women with type 2 diabetes (5/82; 6%) and gestational diabetes (7/82; 9%) were also reported (Table 1).

**TABLE 1:** DKA circumstances, biological features management and outcomes at the time of DKA and according to the type of diabetes. Values are numbers (percentage) unless stated otherwise

	Type 1 diabetes *N* = 70	Type 2 diabetes *N* = 5	Gestational diabetes *N* = 7
Gestational age at diagnosis of DKA (weeks)	*n* = 66	*n* = 5	*n* = 7
First trimester (<13)	8 (12)	0	0
Second trimester (13–27)	13 (20)	1 (20)	1 (14)
Third trimester (28 and beyond)	45 (68)	4 (80)	6 (86)
Suspected aetiology of the DKA	*n* = 70	*n* = 5	*n* = 7
Infection	15 (21)	1 (20)	1 (14)
Medication error	6 (9)	2 (40)	0
Steroid administration	8 (11)	0	3 (43)
Other medication or mechanism	6 (9)	0	0
First diagnosis of diabetes	3 (4)	0	0
Vomiting/hyperemesis	16 (23)	0	1 (14)
Not known	16 (23)	2 (40)	2 (29)
Venous blood glucose level at diagnosis (mmol/L)	*n* = 64	*n* = 5	*n* = 7
Median [Q25,Q75]	14 [11,19]	10 [7,18]	7 [6,11]
<10 mmol/L	14 (22)	2 (40)	5 (71)
10–<14 mmol/L	15 (23)	1 (20)	2 (29)
14–<20 mmol/L	20 (31)	1 (20)	0
≥20 mmol/L	15 (23)	1 (20)	0
Base excess/HCO3^−^ (mmol/L)	*n* = 48	*n* = 5	*n* = 6
Median [Q25,Q75]	11 [6,16]	18 [13,21]	16 [13,17]
<15	34 (71)	2 (40)	2 (33)
Blood ketones (mmol/L)	*n* = 55	*n* = 5	*n* = 5
Median [Q25,Q75]	4.0 [2.4,5.1]	5.1 [3.8, 6.4]	3.8 [1.4, 4.3]
≥3	36 (65)	5 (100)	3 (60)
pH/H+	*n* = 48	*n* = 5	*n* = 5
Median [Q25,Q75]	7.31 [7.23, 7.37]	7.22 [7.14, 7.33]	7.33 [7.30, 7.37]
≤7.10	7 (15)	0	0
Sodium (mmol/L)	*n* = 67	*n* = 5	*n* = 7
<134	36 (54)	3 (60)	2 (29)
Potassium (mmol/L)	*n* = 65	*n* = 5	*n* = 7
<3.5	4 (6)	0	0
>4.5	27 (42)	1 (20)	0
MANAGEMENT			
Highest level of care	*n* = 69	*n* = 5	*n* = 7
Medical/obstetric ward	38 (55)	0	1 (14)
High dependency unit	15 (22)	2 (40)	5 (71)
Intensive care unit	16 (23)	3 (60)	1 (14)
Maternal outcomes			
Hypoglycaemia	*n* = 54	*n* = 4	*n* = 6
	6 (11)	0	1 (17)
Further episodes of DKA	*n* = 66	*n* = 5	*n* = 6
	10 (15)	1 (20)	1 (14)

In three women, the DKA episode was the initial presentation of type 1 diabetes. One woman with type 1 diabetes had a 1^st^ trimester miscarriage at the time of DKA and two women with type 1 diabetes elected to have terminations for personal reasons. Ten percent (7/67) of women with DKA and type 1 diabetes were aged over 35 years, whereas 40% (2/5) and 71% (5/7) of women with type 2 and gestational diabetes, respectively, were over 35.

Women with DKA and type 1 diabetes also had lower body mass index (BMI) at booking with a median (range) BMI of 27 (22–30) kg/m^2^, whereas the women with type 2 or GDM had median BMI of 36 (32–40) kg/m^2^ and 34 (31–37) kg/m^2^, respectively. All five women with DKA and type 2 diabetes were treated with oral glucose‐lowering therapy and two were also treated with insulin prior to pregnancy. Concerning the women with DKA and gestational diabetes, six out of seven had BMIs between 30 and 30.9 kg/m^2^, two women had a previous history of gestational diabetes and one had a history of shoulder dystocia.

### 3.2 Clinical features of the DKA episodes

DKA mainly occurred in the third trimester of pregnancy (55/78; 71%) and was most frequently precipitated by infection (17/82; 21%) and vomiting (17/82; 21%). Steroid therapy in the context of possible preterm birth (11/82; 13%) and medication errors (8/82; 10%) were also frequently reported as causes of DKA. Half of the women (39/76; 51%) had euglycaemic DKA. Twenty‐five percent of women with DKA (20/81) were admitted to an intensive care unit at the time of the episode. No cases of cerebral oedema or aspiration pneumonia were reported. In all, 12 women (15%) had more than one episode of DKA during their pregnancy, and 10 of these women had type 1 diabetes. Two of these women had more than two episodes. Regarding neonatal outcome, 63% of neonates (38/60) were large for gestational age (LGA) and 60% (37/62) were admitted to intensive care.

### 3.3 Case–Control analysis

Out of the population of women with DKA (*N* = 82) three‐quarters had type 1 diabetes. Of the control population (*N* = 280), only 25% had type 1 diabetes. The small numbers of cases of DKA in women with type 2 and gestational diabetes precluded meaningful case–control comparisons in these groups. Therefore, the case–control analysis was confined to women with type 1 diabetes. Data from 70 women with DKA in type 1 diabetes (cases) and 69 women with type 1 diabetes whose pregnancies were not complicated by DKA (controls) were compared (Table 2). Pregnant women with type 1 diabetes who developed DKA and their partners were more likely to be without paid employment at the time of their first antenatal visit (26/70 cases (37%) versus 8/69 controls (12%); OR 4.51 (95% CI: 1.86–10.89)) and were more likely to have a mental health condition (12/70 cases [17%] versus 3/69 controls [5%]; OR 4.55 [95% CI: 1.22–16.9]). Having at least one microvascular complication of diabetes before pregnancy was also associated with DKA (31/70 cases [44%] versus 14/69 controls [14%]; OR 3.12 [95% CI: 1.47–6.63]). Women with DKA who had a caesarean birth were more likely to undergo a caesarean section for maternal compromise (12/37 cases [32%] versus 4/44 controls [9%]; OR 5.75 [95% CI: 1.52–21.73]) and were at greater risk of receiving a general anaesthetic (13/40 cases [32%] versus 4/48 controls [9%]; OR 5.30 [95% CI: 1.56–17.91]). Women with DKA had a higher stillbirth rate (10/62 cases [16%] versus 1/68 controls [1.6%]; OR 12.88 [95% CI: 1.60–103.90]). Among live born infants, the rate of preterm birth (43/52 infants of cases [83%] versus 23/67 infants of controls [34%]; OR 9.14 [95% CI: 3.80–21.99]) and the proportion of neonates admitted to a neonatal unit (34/53 infants of cases [64%] versus 31/67 infants of controls [46%]; OR 2.08 [95% CI: 0.99–4.35]) was higher among infants of cases.

**TABLE 2:** Case–control comparison of maternal characteristics, delivery characteristics and neonatal outcomes in women with type 1 diabetes

	Women with type 1 diabetes and DKA (cases) *N* = 70	Women with type 1 diabetes without DKA (controls) *N* = 69 N = 69
**Maternal characteristics**		
Age, years ≥35	*n* = 67	*n* = 69
	7 (11)	15 (22)
Caucasian ethnicity	*n* = 68	*n* = 69
	63 (93)	63 (91)
Woman and/or partner in paid work at booking	*n* = 70	*n* = 69
	44 (64)	61 (88)
BMI ≥30 kg/m^2^	*n* = 66	*n* = 69
	18 (28)	15 (22)
Median time since diagnosis of diabetes, years [Q25**–**Q75]	*n* = 61	*n* = 56
	15 [9**–**20]	14 [8**–**21]
Any complication of diabetes^a^	*n* = 70	*n* = 69
	31 (44)	14 (20)
Hypertension	*n* = 65	*n* = 62
	12 (19)	11 (18)
Hypothyroidism	*n* = 66	*n* = 62
	13 (20)	8 (13)
Mental health condition	*n* = 70	*n* = 69
	12 (17)	3 (4)
**Pregnancy**		
Woman received pre‐pregnancy counselling	*n* = 45	*n* = 40
	11 (24)	24 (60)
Smoked at any point in pregnancy	*n* = 70	*n* = 69
	22 (31)	13 (19)
Nulliparas	*n* = 68	*n* = 68
	25 (37)	34 (50)
First booking after 12 weeks gestation	*n* = 62	*n* = 69
	8 (13)	8 (12)
Multiple pregnancy	*n* = 69	*n* = 69
	0 (0)	0 (0)
**Birth outcomes**		
Birth induced	*n* = 62	*n* = 68
	22 (36)	30 (44)
Woman laboured	*n* = 61	*n* = 61
	26 (43)	30 (49)
Birth by caesarean section	*n* = 62	*n* = 68
	41 (66)	49 (72)
**Caesarean births**		
Caesarean indication	*n* = 37	*n* = 44
Fetal compromise	13 (35)	17 (39)
Maternal compromise	12 (32)	4 (9)
Other	12 (32)	23 (52)
Caesarean under general anaesthesia	*n* = 40	*n* = 48
	13 (33)	4 (8)
**Neonatal outcomes**		
Stillbirths	*n* = 62	*n* = 68
	10 (16)	1 (2)
Preterm birth <37 weeks gestation^b^	*n* = 52	*n* = 67
	43 (83)	23 (34)
Preterm birth <32 weeks gestation^b^	*n* = 52	*n* = 67
	6 (12)	2 (3)
Large for gestational age (LGA)^b^, ^d^	*n* = 50	*n* = 67
	33 (66)	39 (57)
Small for gestational age (SGA)^b^, ^d^	*n* = 50	*n* = 67
	1 (2)	0 (0)
5 min Apgar <7 ^b^	*n* = 52	*n* = 68
	6 (11)	5 (7)
Infant admitted to ITU^b^	*n* = 52	*n* = 67
	34 (65)	31 (46)
Hypoglycaemia^b^	*n* = 52	*n* = 68
	19 (37)	13 (19)
Respiratory distress syndrome^b^	*n* = 52	*n* = 68
	20 (38)	16 (24)
Jaundice^b^	n = 52	*n* = 68
	15 (29)	17 (25)
Infection^b^	*n* = 52	*n* = 68
	12 (23)	25 (37)
Arterial pH <7,10^c^	*n* = 41	*n* = 38
	9 (22)	6 (16)

Values are numbers (percentage calculated using the population with available data for this variable) unless stated otherwise.

^a^ retinopathy, nephropathy, neuropathy, gastroparesis or microvascular disease.

^b^ % among live births.

^c^ % among children with cord pH at delivery.

^d^ According to Intergrowth curves on weight at birth. Information on birthweight missing for 2 infants.

On multivariable analysis the only factors independently associated with DKA in women with type 1 diabetes were the woman and/or her partner not being in paid employment, adjusted OR 3.64 (95% CI: 1.17–11.39), and having any complication of diabetes, adjusted OR 2.69 (95% CI: 1.05–7.04) (Table 3).

**TABLE 3:** Association between pre‐pregnancy and pregnancy characteristics and DKA in women with type 1 diabetes

	Crude OR (95% CI)	Adjusted OR (95% CI) *N* = 110
Maternal age, years (*n* = 136)		
<35 (*n* = 114)	1	1
**≥**35 (*n* = 22)	0.42 (0.16–1.11)	0.33 (0.11–1.03)
Ethnicity (*n* = 137)		
White (*n* = 126)	1	
Other (*n* = 11)	0.83 (0.24–2.87)	
Woman and/or partner in paid work at booking (*n* = 139)		
Yes (*n* = 105)	1	1
No (*n* = 34)	4.50 (1.86–10.89)	3.64 (1.17–11.39)
BMI (*n* = 135) (kg/m^2)^		
<25 (*n* = 62)	1	
25–30 (*n* = 40)	1.03 (0.46–2.28)	
≥30 (*n* = 33)	1.37 (0.58–3.19)	
Median time since diagnosis of diabetes, years (*n* = 117)		
≤10 (38)	1	1
>10 (79)	1.33 (0.61–2.88)	0.96 (0.37–2.45)
Any complication of diabetes^a^ (*n* = 139)		
No (*n* = 94)	1	1
Yes (*n* = 45)	3.12 (1.47–6.63)	2.69 (1.05–7.04)
Hypertension (*n* = 127)		
No (*n* = 104)	1	
Yes (*n* = 23)	1.05 (0.43–2.59)	
Hypothyroidism (*n* = 128)		
No (*n* = 107)	1	
Yes (*n* = 21)	1.65 (0.63–4.32)	
Mental health condition (*n* = 139)		
No (*n* = 124)	1	1
Yes (*n* = 15)	4.55 (1.22–16.9)	1.69 (0.33–8.60)
Woman received pre‐pregnancy counselling (*n* = 85)		
No (*n* = 50)	1	
Yes (*n* = 35)	0.21 (0.08–0.54)	
Smoked at any point in pregnancy (*n* = 139)		
No (*n* = 104)	1	
Yes (*n* = 35)	1.97 (0.90–4.33)	
Nulliparous (*n* = 136)		
No (*n* = 77)	1	1
Yes (*n* = 59)	0.58 (0.29–1.15)	0.57 (0.24–1.34)
First booking after 12 weeks gestation (*n* = 131)		
No (*n* = 115)	1	1
Yes (*n* = 16)	1.13 (0.40–3.21)	0.50 (0.12–2.10)

^a^ retinopathy, nephropathy, neuropathy, gastroparesis or microvascular disease.

## 4 DISCUSSION

The overall incidence of DKA in pregnancy estimated from this study was 6.3 per 100,000 maternities, affecting approximately 1 in every 60 women with type 1 diabetes giving birth (1.6%) and 1 in every 900 women with type 2 diabetes giving birth (0.11%). DKA in pregnancy did not lead to any maternal deaths but was associated with an exceptionally high perinatal mortality. The stillbirth rate in the population of women with DKA was 160 per 1000 births, compared to the stillbirth rate in the general population in 2018 in the UK of 3.51 per 1000 births^21^ and the overall stillbirth rate from 2014 to 2018 in pregnant women with type 1 or type 2 diabetes of 13.7 per 1000 births.^20^ DKA episodes mostly occurred in women with type 1 diabetes and in the 3^rd^ trimester of pregnancy. Episodes were mainly precipitated by infection, vomiting, steroid therapy and medication errors. Half of the DKA episodes were euglycaemic and 15% of women with DKA episodes had more than one episode in their pregnancy.

The main strength of this study lies in its prospective population‐based design enabling confirmation of the findings from previous, albeit underpowered, studies that perinatal mortality among pregnant women with DKA is high.^22^, ^23^ A previous population‐based study had identified high maternal blood glucose level and high maternal BMI as risk factors for stillbirth in the population with diabetes,^24^ but did not investigate whether or not DKA was a risk factor for stillbirth. Most stillbirths are likely to be caused directly by the DKA episode as intra‐uterine fetal deaths were mainly diagnosed at the time of the episode. Whether it is maternal acidosis, maternal dehydration possibly leading to reduced uteroplacental perfusion, or clinically significant electrolyte imbalance causing fetal arrhythmia, or a combination of these that is responsible for fetal death remains unclear.^10^ As some stillbirths occurred later in relation to the DKA episode, it could also be that DKA in some cases is simply a marker of suboptimal diabetes management, which may cause fetal death by another mechanism. Although first trimester glucose and/or HbA1c data were not available in this study, the high proportion of women with microvascular complications and LGA babies is indicative of suboptimal glycaemic levels both prior to and during pregnancy among those who had DKA. It is reasonable to infer that high perinatal mortality is due to both DKA and a need for better diabetes management, but other intercurrent causes of fetal death cannot be formally ruled out from these data. The size of this national population‐based study was limited by the low incidence of the condition, but we believe it to be the largest contemporary cohort of pregnant women with DKA described. The proportion of missing data is below 10% for most variables and missing data are largely explained by the need for antenatal transfers in cases where DKA episodes were diagnosed and initially managed in one unit and the woman gave birth in another unit.

Although, as would be anticipated, the majority of episodes of DKA occurred in women with type 1 diabetes, women with type 2 and gestational diabetes were also at risk. The small numbers of women with type 2 diabetes and gestational diabetes precluded a case–control analysis. Note that first trimester glucose and/or HbA1c data were not available for the individuals with gestational diabetes, so it is not possible to exclude undiagnosed type 2 diabetes in these women.

One in six women with DKA in pregnancy had a mental health condition. Mental health conditions are a known risk factor for DKA in the non‐pregnant population^25^ and are also associated with poor pregnancy, delivery and neonatal outcomes.^26^, ^27^ Other factors associated with DKA in pregnancy in the present study included smoking at any point in pregnancy and women and/or partner not being in paid work, suggesting that these women were from a more socioeconomically deprived and vulnerable subset of the population with diabetes, which is a known risk factor for DKA in the general population.^25^ The association between established microvascular complications of diabetes and DKA in pregnancy suggests an association not only with difficulties with glucose management but also possibly long‐standing diabetes. More than 10% of the women with DKA experienced at least one further episode during pregnancy, highlighting the need for enhanced diabetes and obstetric specialist support for this vulnerable group.

Episodes of DKA occurred mainly in the third trimester and the reported precipitants included infection, vomiting, steroids and medication error, which is consistent with previous studies.^1^, ^23^ The occurrence of DKA in later pregnancy is likely a manifestation of increasing insulin resistance. This increases the likelihood of DKA in the context of another precipitant that results in absolute or relative insulin deficiency. Infection is generally identified and treated promptly in most pregnancies, but women with type 1 diabetes are at increased risk of infection^28^ and so special emphasis on ‘sick day guidance’ is required, including blood ketone testing. Pregnant women with diabetes are at increased risk of delivering preterm^20^ and antenatal steroids are known to benefit the baby if delivered prematurely. The high proportion of iatrogenic cases of DKA caused by pre‐birth steroids is a concern and should be considered a ‘never event’. Pre‐birth steroids should only be administered under cautious medical surveillance and intensive glucose monitoring in women with diabetes. There is a clear case for national and international guidance on pre‐birth steroids in women with diabetes. Finally, a high number of DKA cases were attributed by clinicians to hyperemesis or vomiting. This may have promoted DKA by prompting inappropriate insulin omission/reduction and by causing dehydration, but alternatively it may also have been a symptom of DKA rather than a precipitant. Overall heightened awareness and support for pregnant women on managing diabetes during any intercurrent illness in pregnancy are required.

Half of DKA episodes were either euglycaemic or associated with only mildly raised blood glucose. Euglycaemia is reported in only 3% of DKA episodes in the general population,^29^ suggesting that DKA occurs more often at lower glucose levels in pregnancy.^14^, ^15^ Various mechanisms have been proposed to explain this occurrence including haemodilution of glucose due to the expanded plasma volume in pregnancy, increased expression of placental glucose transporters and increased glomerular filtration of glucose in pregnancy.^30^

Importantly, half of the women with DKA were managed on general antenatal or medical wards rather than in high‐dependency facilities. Given the requirements for monitoring (both maternal and fetal), the high prevalence of metabolic disturbance and the substantial risks of stillbirth, the optimal place to manage pregnant women with DKA needs to be carefully considered.

## 5 CONCLUSION

DKA in pregnancy was associated with high perinatal mortality and was linked with factors related to socio‐economic deprivation, mental illness and microvascular complications. Guidelines are needed on pre‐pregnancy care as well as the optimum management of DKA in pregnancy (including location of care) and on appropriate obstetric/diabetes management following episodes of DKA, given the high risk of recurrent DKA, preterm birth and stillbirth.

## ETHICS

This study was approved by the North London Research Ethics Committee 1 (Ref. Number of approval 10/H0717/20).

## CONFLICT OF INTEREST

The authors have declared that no competing interests exist.

## References

[1] Kilvert JA , Nicholson HO , Wright AD . Ketoacidosis in diabetic pregnancy. Diabet Med. 1993;10(3):278‐281. doi:10.1111/j.1464-5491.1993.tb00059.x 8387415

[2] Chauhan SP , Perry KG , McLaughlin BN , Roberts WE , Sullivan CA , Morrison JC . Diabetic ketoacidosis complicating pregnancy. J Perinatol. 1996;16(3 Pt 1):173‐175. 8817424

[3] Bryant SN , Herrera CL , Nelson DB , Cunningham FG . Diabetic ketoacidosis complicating pregnancy. J Neonatal Perinatal Med. 2017;10(1):17‐23. doi:10.3233/NPM-1663 28304323

[4] Centers for Disease Control and Prevention . Maps of trends in diagnosed diabetes and obesity. Published online April 27, 2017. https://www.cdc.gov/diabetes/statistics/slides/maps_diabetesobesity_trends.pdf

[5] Mayer‐Davis EJ , Lawrence JM , Dabelea D , et al. Incidence trends of type 1 and type 2 diabetes among youths, 2002–2012. N Engl J Med. 2017;376(15):1419‐1429. doi:10.1056/NEJMoa1610187 28402773 PMC5592722

[6] Mackin ST , Nelson SM , Kerssens JJ , et al. Diabetes and pregnancy: national trends over a 15 year period. Diabetologia. 2018;61(5):1081‐1088. doi:10.1007/s00125-017-4529-3 29322220 PMC6448996

[7] Bell R , Bailey K , Cresswell T , Hawthorne G , Critchley J , Lewis‐Barned N . Trends in prevalence and outcomes of pregnancy in women with pre‐existing type I and type II diabetes. BJOG. 2008;115(4):445‐452. doi:10.1111/j.1471-0528.2007.01644.x 18271881

[8] Kamalakannan D , Baskar V , Barton DM , Abdu TAM . Diabetic ketoacidosis in pregnancy. Postgrad Med J. 2003;79(934):454‐457. doi:10.1136/pmj.79.934.454 12954957 PMC1742779

[9] Frise CJ , Mackillop L , Joash K , Williamson C . Starvation ketoacidosis in pregnancy. Eur J Obstet Gynecol Reprod Biol. 2013;167(1):1‐7. doi:10.1016/j.ejogrb.2012.10.005 23131345

[10] Sibai BM , Viteri OA . Diabetic ketoacidosis in pregnancy. Obstet Gynecol. 2014;123(1):167‐178. doi:10.1097/AOG.0000000000000060 24463678

[11] Parker JA , Conway DL . Diabetic ketoacidosis in pregnancy. Obstet Gynecol Clin North Am. 2007;34(3):533‐543, xii. doi:10.1016/j.ogc.2007.08.001 17921013

[12] Ramin KD . Diabetic ketoacidosis in pregnancy. Obstet Gynecol Clin North Am. 1999;26(3):481‐488, viii. doi:10.1016/s0889-8545(05)70092-9 10472067

[13] Dalfrà MG , Burlina S , Sartore G , Lapolla A . Ketoacidosis in diabetic pregnancy. J Matern Fetal Neonatal Med. 2016;29(17):2889‐2895. doi:10.3109/14767058.2015.1107903 26461169

[14] Guo R‐X , Yang L‐Z , Li L‐X , Zhao X‐P . Diabetic ketoacidosis in pregnancy tends to occur at lower blood glucose levels: case‐control study and a case report of euglycemic diabetic ketoacidosis in pregnancy. J Obstet Gynaecol Res. 2008;34(3):324‐330. doi:10.1111/j.1447-0756.2008.00720.x 18588610

[15] Darbhamulla S , Shah N , Bosio P . Euglycaemic ketoacidosis in a patient with gestational diabetes. Eur J Obstet Gynecol Reprod Biol. 2012;163(1):118‐119. doi:10.1016/j.ejogrb.2012.03.011 22465827

[16] Madaan M , Aggarwal K , Sharma R , Trivedi SS . Diabetic ketoacidosis occurring with lower blood glucose levels in pregnancy: a report of two cases. J Reprod Med. 2012;57(9–10):452‐455. 23091997

[17] de Veciana M . Diabetes ketoacidosis in pregnancy. Semin Perinatol. 2013;37(4):267‐273. doi:10.1053/j.semperi.2013.04.005 23916025

[18] Joint British Diabetes Societes for inpatient care . Intravenous Insulin Prescription and Fluid Protocol for diabetic keto‐acidosis. https://abcd.care/sites/abcd.care/files/resources/2018_addition_DKA_IPC_Pathway.pdf

[19] Villar J , Ismail LC , Victora CG , et al. International standards for newborn weight, length, and head circumference by gestational age and sex: the Newborn Cross‐Sectional Study of the INTERGROWTH‐21st Project. Lancet. 2014;384(9946):857‐868. doi:10.1016/S0140-6736(14)60932-6 25209487

[20] National Pregnancy in Diabetes (NPID) Audit Report 2018 England, Wales and the Isle of Man. https://files.digital.nhs.uk/CF/4791D9/National%20Pregnancy%20in%20Diabetes%20Audit%20Report%202018.pdf

[21] Draper ES , Gallimore ID , Smith LK , … et al.; on behalf of the MBRRACE‐UK Collaboration . MBRRACE‐UK Perinatal Mortality Surveillance Report, UK Perinatal Deaths for Births from January to December 2018. The Infant Mortality and Morbidity Studies, Department of Health Sciences, University of Leicester; 2020.

[22] Schneider MB , Umpierrez GE , Ramsey RD , Mabie WC , Bennett KA . Pregnancy complicated by diabetic ketoacidosis: maternal and fetal outcomes. Diabetes Care. 2003;26(3):958‐959. doi:10.2337/diacare.26.3.958 12610076

[23] Cullen MT , Reece EA , Homko CJ , Sivan E . The changing presentations of diabetic ketoacidosis during pregnancy. Am J Perinatol. 1996;13(7):449‐451. doi:10.1055/s-2007-994386 8960616

[24] Mackin ST , Nelson SM , Wild SH , Colhoun HM , Wood R , Lindsay RS . Factors associated with stillbirth in women with diabetes. Diabetologia. 2019;62(10):1938‐1947. doi:10.1007/s00125-019-4943-9 31353418 PMC6731193

[25] Ehrmann D , Kulzer B , Roos T , Haak T , Al‐Khatib M , Hermanns N . Risk factors and prevention strategies for diabetic ketoacidosis in people with established type 1 diabetes. Lancet Diabetes Endocrinol. 2020;8(5):436‐446. doi:10.1016/S2213-8587(20)30042-5 32333879

[26] Jablensky AV , Morgan V , Zubrick SR , Bower C , Yellachich L‐A . Pregnancy, delivery, and neonatal complications in a population cohort of women with schizophrenia and major affective disorders. Am J Psychiatry. 2005;162(1):79‐91. doi:10.1176/appi.ajp.162.1.79 15625205

[27] Nguyen TN , Faulkner D , Frayne JS , et al. Obstetric and neonatal outcomes of pregnant women with severe mental illness at a specialist antenatal clinic. Med J Aust. 2013;199(3 Suppl):S26‐29. doi:10.5694/mja11.11152 25369845

[28] Carey IM , Critchley JA , DeWilde S , Harris T , Hosking FJ , Cook DG . Risk of infection in type 1 and type 2 diabetes compared with the general population: a matched cohort study. Diabetes Care. 2018;41(3):513‐521. doi:10.2337/dc17-2131 29330152

[29] Jenkins D , Close CF , Krentz AJ , Nattrass M , Wright AD . Euglycaemic diabetic ketoacidosis: does it exist? Acta Diabetol. 1993;30(4):251‐253. doi:10.1007/BF00569937 8180418

[30] Bonora BM , Avogaro A , Fadini GP . Euglycemic Ketoacidosis. Curr Diab Rep. 2020;20(7):25. doi:10.1007/s11892-020-01307-x 32424730

